# Advanced External Beam Stereotactic Radiotherapy for Skull Base Reirradiation

**DOI:** 10.3390/cancers17030540

**Published:** 2025-02-05

**Authors:** He Wang, Fahed M. Alsanea, Dong Joo Rhee, Xiaodong Zhang, Wei Liu, Jinzhong Yang, Zhifei Wen, Yao Zhao, Tyler D. Williamson, Rachel A. Hunter, Peter A. Balter, Tina M. Briere, Ronald X. Zhu, Anna Lee, Amy C. Moreno, Jay P. Reddy, Adam S. Garden, David I. Rosenthal, Gary B. Gunn, Jack Phan

**Affiliations:** 1 Radiation Physics, University of Texas M.D. Anderson Cancer Center, Houston, TX 77030, USA; fmalsanea@mdanderson.org (F.M.A.); drhee1@mdanderson.org (D.J.R.); xizhang@mdanderson.org (X.Z.); jyang4@mdanderson.org (J.Y.); yzhao15@mdanderson.org (Y.Z.);; 2 Medical Physics, Mayo Clinic College of Medicine and Science, Phoenix, AZ 85054, USA; 3 Radiation Oncology, Hoag Memorial Hospital, Hoag Cancer Center, Newport Beach, CA 92663, USA; 4 Radiation Therapeutic Physics, University of Texas M.D. Anderson Cancer Center, Houston, TX 77030, USA; 5 Radiation Oncology, University of Texas M.D. Anderson Cancer Center, Houston, TX 77030, USAjphan@mdanderson.org (J.P.)

**Keywords:** stereotactic body radiation therapy, reirradiation, skull base cancer, external beam radiation therapy, Gamma Knife, CyberKnife, proton therapy, dose gradient, intensity-modulated proton therapy, volumetric modulated radiation therapy

## Abstract

Background/Objectives: Stereotactic body radiation therapy (SBRT) for skull base reirradiation is particularly challenging, as patients have already received substantial radiation doses to the region, and nearby normal organs may have approached their tolerance limit from prior treatments. In this study, we reviewed the characteristics and capabilities of four advanced external beam radiation delivery systems and four modern treatment planning systems and evaluated the treatment plan quality of each technique using skull base reirradiation patient cases. Methods: SBRT plans were generated for sixteen skull base reirradiation patients using four modalities: the GK plan for the Elekta Leksell Gamma Knife Perfexion/ICON, the CyberKnife (CK) plan for the Accuray CyberKnife, the intensity-modulated proton therapy (IMPT) plan for the Hitachi ProBeat-FR proton therapy machine, and the volumetric-modulated arc therapy (VMAT) plan for the Varian TrueBeam STx. These plans were evaluated and compared using two novel gradient indices in addition to traditional dosimetry metrics for targets and organs at risk (OARs). The steepest border gradient quantified the percent prescription dose fall-off per millimeter at the boundary between the target and adjacent critical structures. This gradient index highlighted the system’s ability to spare nearby critical OARs. The volume gradient assessed the extent of dose spread outside the target toward the patient’s body. Results: All plans achieved comparable target coverage and conformity, while IMPT and VMAT demonstrated significantly better uniformity. The GK plans exhibited the highest border gradient, up to 20.9%/mm, followed by small-spot-size IMPT plans and CK plans. Additionally, IMPT plans showed the benefit of reduced dose spread in low-dose regions and the lowest maximum and mean doses to the brainstem and carotid artery. Conclusions: The advanced external beam radiotherapy modalities evaluated in this study are well-suited for SBRT in skull base reirradiation, which demands precise targeting of tumors with highly conformal doses and steep dose gradients to protect nearby normal structures.

## Simple Summary

Skull base stereotactic radiotherapy (RT) is particularly challenging due to prior radiation and the proximity of several critical organs. This study reviewed four advanced external beam RT modalities and their corresponding or available modern treatment planning systems (TPSs). The plan quality and potentials were evaluated in terms of target coverage and dose gradient. The steepest border gradient was used to assess the fall-off speed achievable near the target to spare adjacent critical structures, while the volume gradient was used to evaluate dose spread at a distance. Gamma Knife demonstrated the highest border gradient, followed by small-spot-size proton beams and CyberKnife. The proton beam exhibited the least dose spread in the low-dose region.

## 1. Introduction

The treatment of skull base cancer is complex and usually presents unique challenges due to the intricate anatomy and the proximity of various critical structures, such as the brainstem, optic apparatus, major vessels, and numerous cranial nerves. Typically, a multidisciplinary approach is required, involving a combination of surgery, radiation therapy, and sometimes chemotherapy or targeted therapies, to improve local control and survival rates [1,2,3,4,5,6,7].

Radiation therapy (RT) is commonly employed in the treatment of skull base cancers for patients who are surgically unresectable, have residual tumors following surgical resection, or possess physical or medical conditions that pose a high risk for surgery. Notably, stereotactic radiation therapy (SBRT) has emerged as an attractive option for localized residual or recurrent tumors [8,9,10]. SBRT delivers highly conformal ablative radiation in a small number of fractions over a short time period, typically within two weeks, in contrast to the 6–7 weeks required for conventional head and neck cancer treatment.

Reirradiation of skull base recurrences is among the most challenging cases as the patients have already received a significant radiation dose to the same region, and surrounding normal organs may have reached their tolerance dose from prior RT [11,12]. Several modern external beam radiation delivery systems are used for SBRT treatment to provide precise targeting of tumors with highly conformal doses and steep dose gradients, thereby minimizing damage to surrounding healthy tissues. These advanced systems include Gamma Knife (Elekta Instruments AB, Stockholm, Sweden) [13,14], CyberKnife (Accuray, Inc., Sunnyvale, CA, USA) [15,16,17], linear accelerators (LINACs) [18], Tomotherapy (TomoTherapy Inc., Madison, WI, USA) [19,20], and proton therapy machines [21,22]. Despite their differences in design, radiation sources, and delivery strategies, these systems all provide effective SBRT treatment.

In addition to the precise targeting requirements, treatment planning systems (TPSs) face similar challenges, particularly in ensuring dose calculation accuracy for small fields and providing the optimization tools necessary to achieve dosimetric goals [23,24,25]. The combination of a high-precision radiation delivery system and an optimal planning solution is crucial to achieving optimal treatment outcomes while minimizing toxicities.

Recent studies have demonstrated comparable overall survivals and relatively lower incidences of severe toxicities with SBRT in the reirradiation setting compared to conventional fractionated radiation therapy [26,27]. To achieve optimal sparing of organs at risk (OARs) without compromising target coverage, much stricter dose constraints are typically employed during the treatment planning process. In this context, the dose gradient at the boundary between the target and adjacent critical structures plays a pivotal role in the SRS/SRT/SBRT field. However, the traditionally used gradient index—commonly defined as the ratio of the 50% isodose line volume to the 100% isodose line volume [28]—does not adequately capture the sharpness of dose fall-off and its spatial relationship to critical structures. This limitation underscores the need for more refined metrics to evaluate dose gradients, particularly in complex treatment scenarios involving close proximity of targets to critical structures.

While numerous vendors and modalities of radiation treatment machines and various treatment planning systems are available, this study focused on reviewing the SBRT capabilities of a select group of advanced external beam RT delivery modalities and RT treatment planning systems, and investigated their dosimetric potentials, including novel gradient metrics, using skull base SBRT cases.

## 2. Materials and Methods

The outcomes of external beam radiation treatment are closely correlated with the delivery systems and treatment planning systems. For skull base SBRT, the requirements of these systems play a pivotal role in achieving precise targeting while maximizing normal tissue sparing. Before conducting the comparison of achievable treatment plans, we summarized the characteristics side-by-side in tables for the systems that were used in this study. The information is primarily sourced from vendor specifications.

### 2.1. External Beam Radiation Delivery Systems

The requirements for an external beam delivery system used for SBRT include mechanical and radiation accuracy, small-field collimation, volumetric imaging capability, and treatment efficiency. Table 1 compares the features and capabilities of four advanced systems used in this study: Elekta Leksell Gamma Knife Perfexion/ICON, Accuray CyberKnife M6/S7, Hitachi ProBeat-FR (representative of the proton therapy machines), and Varian TrueBeam STx (representative of LINAC machine).

LINACs are the most used external beam radiation therapy modality due to their versatility in radiation techniques, beam energies, and dose rates, enabling the treatment of various types and locations of cancer [42,43]. The TrueBeam STx is one of the premier LINAC machines, and it is highly regarded for its capabilities in SBRT. The Gamma Knife is specifically designed with a head frame to treat small intracranial lesions with high precision in single-fraction stereotactic radiosurgery (SRS). The advanced ICON version integrates cone-beam computed tomography (CBCT) and motion management, allowing for frameless and fractionated treatment. The CyberKnife is a robotic system capable of delivering radiation beams from nearly any angle, with real-time imaging for motion tracking. Both Gamma Knife and CyberKnife are dedicated SRS/SBRT machines. Proton beam therapy is renowned for its “Bragg peak”, which allows energy deposition within the tumor while protecting surrounding healthy tissue and organs with no exit dose. The Hitachi ProBeat-FR, with its small spot sizes, is well-suited for SBRT.

Table 1 also lists the references for commissioning and quality assurance of these machines. The test and tolerances must adhere to the recommendations for the SRS/SBRT procedures.

Image guidance is the key element in stereotactic RT to enhance the precision and accuracy of radiation delivered to target while minimizing exposure to the surrounding healthy tissues. Fast kV volumetric imaging, such as cone-beam CT (CBCT), is the typical onboard imaging system on radiation modalities, as seen in Gamma Knife ICON, TrueBeam, and ProBeat. However, these systems lack the capability for real-time tracking. CyberKnife does not use CBCT technology; instead, it employs advanced live X-ray imaging that can continuously track the target during treatment and can perform real-time adaptations to compensate for patient motion. In addition to onboard imaging systems, several advanced technologies can be integrated with radiation delivery systems to facilitate efficient and accurate patient setup verification as well as motion tracking. These include BrainLab Exactrac systems (X-ray and surface tracking) [44,45], CT-on-rail imaging [46], and surface guidance systems [47,48,49] such as VisionRT, C-Rad, etc. Modern techniques have recently emerged that integrate MRI and PET with LINACs, providing superior visualization of tumors at functional level [50,51], allowing biology-guided radiotherapy [52,53], and helping to improve radiation treatment outcomes.

### 2.2. Radiation Treatment Planning Systems

The treatment planning system is also crucial for generating high-precision SBRT plans to ensure effective and safe skull base cancer treatment. Corresponding to the RT delivery systems listed in Table 1, we compare the features and capabilities of four TPS systems in Table 2: the Leksell GammaPlan® for Gamm Knife Perfexion/ICON (GK), the Accuray Precision® for CyberKnife (CK), and RayStation® (RaySearch Laboratories) for both proton ProBeat and TrueBeam STx modalities. Plans from these four systems will be evaluated for our skull base reirradiation cases.

In summary, GammaPlan and Accuray Precision are the dedicated TPSs designed for Leksell Gamm Knife and CyberKnife, respectively, both specializing in non-isocentric treatment planning for SRS/SBRT patients. RayStation TPS supports multiple treatment modalities and offers several powerful tools that make it a superior choice for external beam radiation therapy. These include a multi-criteria optimization tool that helps users understand the tradeoffs between conflicting objectives using Pareto planes, an adaptive planning tool that can enhance the efficiency of adaptive treatment workflow, robust optimization that is particularly beneficial for particle beam therapy, and an advanced scripting tool that facilitate the automation of processing.

Many additional capabilities common to all these TPS include multi-modality imaging fusion, inverse planning, non-coplanar beam geometry, and dose-volume histogram (DVH) analysis.

### 2.3. Patients and Treatment Plan Generation

Sixteen patients who underwent SBRT for skull base reirradiation on IRB-approved trials (SOAR 2016-1065; PA14-0198) were randomly selected. Nine patients received treatment on Varian TrueBeam STx with a prescription dose (Rx) of 45 Gy delivered in 5 fractions. Seven patients were treated on Elekta Leksell Gamma Knife Perfexion, receiving prescription doses ranging from 21 Gy to 27 Gy in 3 fractions. The mean initial treatment prescription was 66 Gy (range: 60 to 70 Gy) in 30–33 fractions. The mean reirradiation interval was 23 months (range: 3 to 57 months). Table 3 presents detailed patient and SBRT treatment information, with the primary target volume ranging from 2.1 cm^3^ to 36.4 cm^3^.

Treatment plans were generated using the four TPSs in Table 2 for treatment machines specified in Table 1. Identical target volumes were used for planning consistency. The proton plans were generated in RayStation R12A for Hitachi ProBeat-FR utilizing 3 to 5 non-coplanar beams with intensity modulation proton therapy (IMPT) technique and the Monte Carlo dose calculation engine. Robust optimization was applied with a 2 mm setup uncertainty and 2.5% range uncertainty. The volumetric modulated arc therapy (VMAT) plans were generated in RayStation R11A with 2 to 3 arcs for Varian TrueBeam STx using the collapsed cone convolution dose calculation engine. GK plans were manually created in Leksell Gamma Plan 10.1 for Leksell Gamma Knife Perfexion, while CyberKnife plans were created in Accuray Precision for the CyberKnife M6 employing multi-leaf collimators (MLC) and Monte Carlo dose calculation engine. All plans were generated by experienced medical physicists or dosimetrists.

The planning goals aimed to achieve comparable or improved target coverage while adhering to clinical dose constraints for critical organs or structures. The general clinical goals for 3- and 5-fraction reirradiation treatment plans are listed in Table 4. The constraints for OARs were much stricter than those for conventional treatment due to reirradiation. Evaluation includes comparing the target coverage, Paddick conformity index (PCI) [57], and homogeneity index (HI). HI is calculated as (D2-D98)/D50, where Dx represents the dose to x% of the volume in cumulative DVH. PCI values are ≤1.0, with 1.0 indicating perfect conformity; HI values are ≥0.0, with 0.0 indicating perfect uniformity.

Traditional dose gradient analysis typically employs a single gradient index, calculated as the ratio of volume enclosed by the 50% Rx isodose line (IDL) to the volume enclosed by 100% IDL [28]. In skull base SBRT, multiple critical OARs may be in close proximity to or overlapping with the radiation target, necessitating a balance between the target coverage and OAR sparing. Of particular interest is the speed of dose fall-off at the boundary, which is crucial for estimating or predicting the target coverage versus normal tissue sparing. Given the use of different radiation sources and collimations across RT modalities, the rate of dose fall-off may vary. Therefore, our study is designed to evaluate two new gradient indices, both as functions of percent prescription dose (%Rx), enabling their application across different prescribed doses.

Steepest border gradient (SBG). SBG is defined as the highest percent of Rx dose fall-off per mm (%/mm) at the %Rx isodose line. It serves to evaluate the rapidity of dose fall-off at the boundary between the target and adjacent critical structures. An in-house developed script was employed to detect the shortest distance from the x% prescription isodose line IDL (%Rx) to the prescription isodose line IDL (Rx). Distances to IDL (Rx) were recorded for isodose lines ranging from 50% to 90% of the prescription dose; then, they were converted to %/mm.

Volume gradient (VG). VG is defined as VOL (%Rx)/TV, where VOL (%Rx) represents the volume enclosed by the IDL (%Rx), and TV is the volume of the primary target. VG assesses the speed of dose-volume spread-out, which is pertinent to the integral dose considerations. VG values were recorded from the 100% Rx IDL down to the 20% Rx IDL. The value of VG at 100% Rx IDL correlates with the RTOG conformity index [58], while the traditional gradient index [28] can be derived from VG values at 50% Rx IDL and 100% Rx IDL.

Both the SBG and VG are influenced by factors such as beam penumbra, beam angle arrangement, and the optimization constraints controlling the gradient-related parameters. These metrics will be compared across plans generated for the four RT modalities.

The maximum dose (defined as the dose to hottest 0.01 cm^3^) and mean dose to the brainstem and the ipsilateral carotid arteries were recorded and normalized to the prescription dose. These metrics were compared among the RT plans. The brainstem and carotid arteries are among the most critical OARs in the majority of skull base reirradiation cases.

The above plan quality metrics were evaluated and compared among the four treatment plans. All plans were normalized to meet similar dose constraints on critical OARs while achieving best possible target coverage. Statistical analysis was performed using the Wilcoxon signed-rank test for comparison in IBM SPSS Statistics 24. A *p*-value < 0.05 was considered statistically significant.

## 3. Results

**Figure 1:**
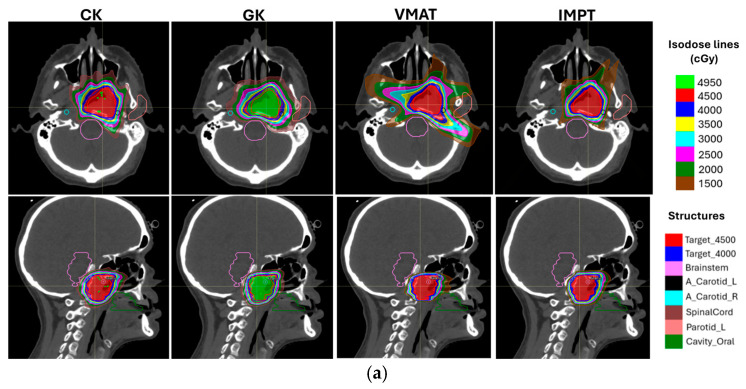

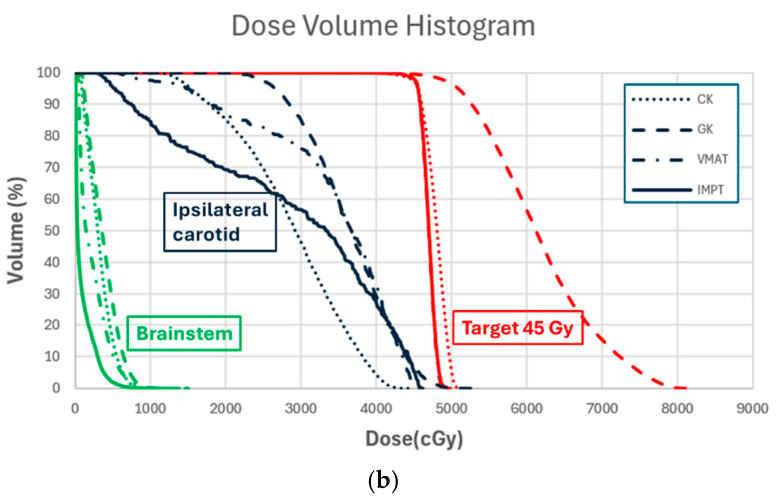
A representative case showing SBRT plans from CyberKnife (CK), Gamma Knife (GK), intensity-modulated proton therapy (IMPT), and volumetric modulated arc therapy (VMAT) techniques. The patient initially received 70 Gy in 33 fractions in 2013 and underwent a VMAT SBRT for left nasopharynx recurrence in 2015 (20-month intervals). (**a**) The transverse view (top row) and sagittal view (bottom row) of the plan dose distributions. Several organs at risk surround the target, and the plans were generated to meet clinical goals outlined in Table 4. (**b**) Dose-volume histograms of the primary target, brainstem, and ipsilateral carotid for the same patient.

The delivery systems listed in Table 1 are well-suited for SBRT in skull base reirradiation, enabling precise targeting. The treatment planning systems outlined in Table 2 can achieve adequate dose gradients to spare adjacent critical structures through effective optimization. Figure 1 illustrates the dose distributions of representative SBRT plans generated using CK, GK, VMAT, and IMPT techniques.

**Figure 2:**
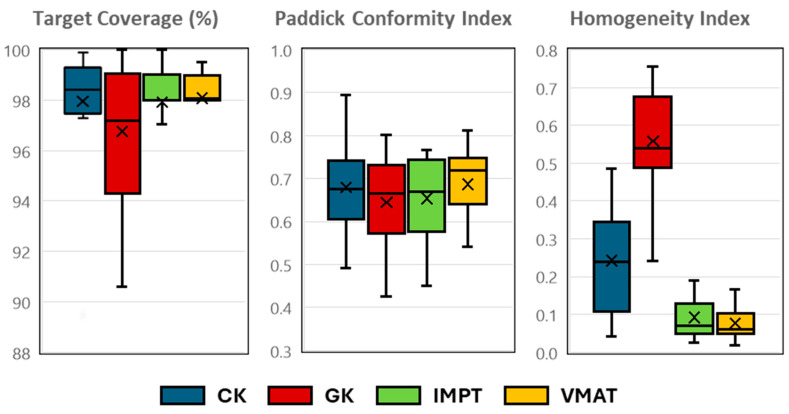
Comparison of primary target coverage, Paddick conformity index, and homogeneity index for CyberKnife (CK), Gamma Knife (GK), intensity-modulated proton therapy (IMPT), and volumetric modulated arc therapy (VMAT).

Figure 2 displays the target coverage, PCI, and HI for the primary target of all 16 patients. The target coverage and PCI show comparable results across all four plans (*p* > 0.05). However, the HI is notably lower for IMPT and VMAT plans compared to CK and GK plans (*p* < 0.01), indicating superior uniformity in dose distribution for IMPT and VMAT.

**Figure 3:**
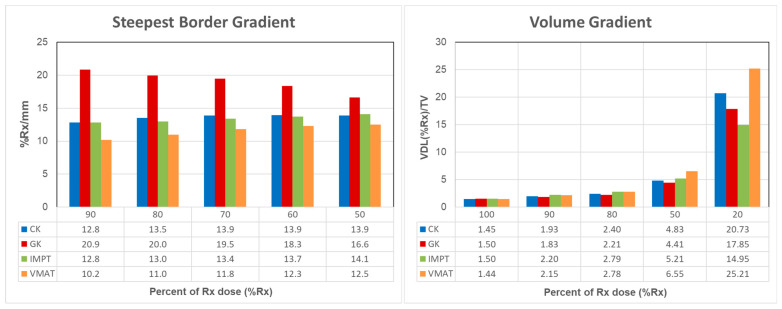
Comparison of the steepest border gradient (**left**) and volume gradient (**right**) for CyberKnife (CK) Gamma Knife (GK), intensity-modulated proton therapy (IMPT), and volumetric modulated arc therapy (VMAT).

Figure 3 compares the SBG and VG among the four plans. Given the stringent brainstem dose constraints in skull base reirradiation, the highest dose gradient typically occurred at the boundary between the brainstem and the target. Analysis of the SBG reveals that for GK plans, the first 10% dose fall-off occurred within approximately 0.5 mm, resulting in an SBG as high as 20.9%/mm (Figure 3(left)). In contrast, CK, IMPT, and VMAT plans showed a 10% dose fall-off within about 1 mm, corresponding to 10.2%/mm to 12.8%/mm. At the 50% Rx isodose line, the mean fall-off speed decreased to 16.6% for GK plans, while the other three plans showed an increase. This variation is linked to plan normalization: GK plans typically prescribe 50% of the maximum dose, positioning the Rx isodose line at the steepest gradient of the dose profile. In contrast, VMAT and IMPT plans often prescribe to the shoulder (90% or above) of the dose profile, placing the steepest gradient at a lower isodose line location. Overall, GK plans showed the highest SBG compared to the other three techniques (*p* < 0.05) between 100% and 50% Rx IDLs. A 50% dose drop occurred in approximately 3 mm for the GK plan, compared to around 4 mm for IMPT, CK, and VMAT plans.

Figure 3 (right) illustrates the volume gradient comparison among the four techniques. At the 100% Rx isodose line, GK and IMPT plans covered a larger volume. As distances increased from the target, CK and VMAT plans showed an increase in volume for the 50% Rx isodose line, whereas IMPT maintained a larger volume than GK. By the 20% Rx isodose line, IMPT plans exhibited significantly lower volumes compared to the other techniques (*p* < 0.05).

**Figure 4:**
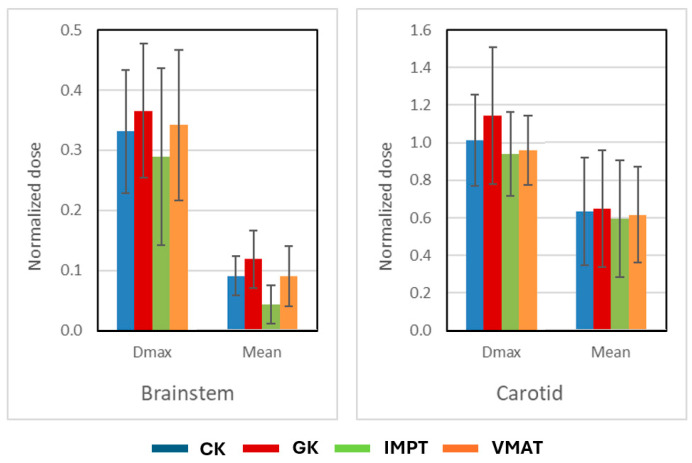
Comparison of the brainstem (**left**) and carotid (**right**) dose with one standard deviation for CyberKnife (CK), Gamma Knife (GK), intensity-modulated proton therapy (IMPT), and volumetric modulated arc therapy (VMAT). Doses are normalized to prescription doses.

Figure 4 compares the OAR doses among the four treatment plans. The brainstem was within 5 mm distance of the target for 9 out of 16 patients and within 2 mm for 4 out of 16 patients. The ipsilateral carotid artery partially overlapped with the target in 12 out of 16 patients and was within 2 mm of the target in 3 out of 16 patients. Due to the stringent dose constraints for the brainstem, plans typically exhibited sharp dose gradients toward it. Comparing the four techniques, IMPT plans demonstrated superior maximum and mean doses to the brainstem (*p* < 0.05). The mean dose to the ipsilateral carotid artery was comparable across all techniques, but the CK, IMPT, and VMAT plans achieved better adherence to the clinical goal of avoiding hot spots within the carotid artery compared to the GK plans (*p* < 0.05).

The comparison of the above plan quality metrics is also summarized in Table 5. Table 5 also compared the beam-on-time and delivery time across the four treatment techniques. Notably, for CK and GK, the beam-on-time and delivery time were identical, as they continuously delivered all shots or beamlets. For IMPT and VMAT plans, the delivery time encompasses the duration from initiating the first beam to completing all beams, including time for field changes and verification, image acquisition before each beam, and couch rotation for non-coplanar beams. The beam-on-time calculations were based on specific delivery dose rates: 3 Gy/min for GK and 600 MU/min for VMAT plans.

## 4. Discussion

Skull base recurrences are associated with high mortality rates and severe morbidity due to the local destruction of surrounding critical organs [11]. Typically, reirradiation is the only viable option for unresectable recurrences. However, reirradiation of the skull base tumors presents significant challenges. The proximity of critical structures, including the brainstem, spinal cord, optic apparatus, cochlea, major vessels, and numerous cranial nerves, increases the risk of severe radiation-induced toxicities. These structures may have already received a dose close to tolerance in the initial radiation therapy, which is typically 60–70 Gy in 30–35 fractions. Moreover, the separations between the tumors and vital structures may be only submillimeter, which necessitates an intricate balance between delivering a sufficient tumoricidal dose and sparing multiple critical structures, each with distinct dose tolerances. These complexities underscore the importance of advanced techniques and precise radiation delivery systems to minimize the risk of devastating outcomes while maximizing treatment efficacy.

In this study, we compared four external beam radiation techniques for SBRT with those used for skull base reirradiation. As shown in Table 4, the dose constraints were significantly stricter for reirradiation to minimize post-radiation complications. Specifically, we introduced a novel gradient concept to evaluate and compare the performance of these techniques in skull base SBRT and identified the potential gradient each technique could achieve in terms of OAR sparing. The steepest border gradient, expressed as the sharpest dose fall-off speed (%Rx/mm), evaluates each system’s ability to achieve the steepest dose gradient when critical structures are near or adjacent to the target. This metric is particularly valuable in balancing target coverage with OAR sparing. It provides essential guidance during the treatment planning process by defining achievable planning goals for each system. Additionally, it offers a deeper understanding of the dosimetric consequences of daily patient setup errors.

Complementing the steepest border gradient, the volume gradient, which is similar to the traditional gradient index, was utilized to assess the dose spread throughout the patient’s body outside the target volume. This measure further aids in understanding the overall dose distribution and minimizing unnecessary radiation exposure to healthy tissues.

For the other commonly used metrics for plan evaluation, all techniques achieved comparable target coverage and conformity, while VMAT and IMPT demonstrated superior homogeneity. LINAC-based SBRT emerged as the most utilized technique due to its versatility in treating various cancer types and its specific features that are well-suited for SBRT. However, the dose spread was highest in VMAT plans, which necessitates careful design before planning to minimize unintended dose exposure to healthy tissues, especially when non-coplanar arcs are used.

GK demonstrated the most effective capability in creating the steepest immediate dose fall-off at the boundary of the target, thereby sparing critical structures that are proximal or abutting. Following GK, IMPT also showed significant benefits in limiting dose spread beyond the 50% Rx isodose line. However, the efficacy of proton treatment depends on the target location and the number of beams utilized. In our study, proton beams utilized extended-range shifters located closer to the patient for more superficial targets, without the use of apertures, which are known to further reduce dose spread both locally and at a distance, according to references [59,60,61,62]. The larger volume observed for the isodose lines from 100% to 50% in IMPT plans was influenced by robust optimization techniques employed during planning, which also impacted the CI for IMPT. Typically, a 2 mm margin was used to account for patient position uncertainty in robust optimization. Online daily adaptation strategies may help reduce this uncertainty through daily imaging and provide additional benefits by minimizing radiation exposure to surrounding tissues.

The size of the proton spot is crucial for stereotactic radiation therapy. In our previous study, we utilized IMPT for head and neck SBRT using an early version of ProBeat, which had a spot size greater than 1 cm [63]. Plans with larger spot sizes did not demonstrate clear benefits in target coverage and gradient enhancement typical of proton treatment. However, the current Probeat-FR system features a spot size of approximately 5 mm, making it suitable for stereotactic treatment. The smaller spot size improves the proton system’s ability to achieve a steep border gradient, with optimal volume gradient observed outside the 50% Rx isodose line as well.

While the primary focus of this study is not on dose calculation algorithms, the dosimetry accuracy of the TPSs must be meticulously assessed for small-field radiation [64], especially in the region of a high dose gradient. Monte Carlo-based dose calculation is renowned for its widely accepted accuracy, and it has overcome the computational time through the use of GPUs in RayStation for proton plans [65,66]. The VMAT plans generated in RayStation for this study employ the collapsed cone-based algorithm, which has demonstrated accuracy within 3% [67,68,69] for small-field irradiation. The TPSs for GK and CK are specially designed for SRS/SBRT. CK utilizes Monte Carlo dose calculation for MLC-based plans, while GK requires a CT scan to use a convolution algorithm to account for heterogeneity in dose calculation [34,70]. It is crucial to note that the accuracy of dose calculation in these TPSs is contingent upon the precision of beam modeling during the TPS commissioning. This accuracy should be thoroughly validated through comprehensive end-to-end testing, with particular attention to small field scenarios [71].

We used the same target volumes across all techniques in this study, applying a 2 mm margin to skull base lesions based on our patient setup protocol [72]. Similar immobilization techniques were assumed for the four techniques evaluated, facilitating hypofractionated treatment for skull base reirradiation. Additionally, some patients had subclinical risk target volumes contoured around the primary target to receive lower doses aimed at covering sites of potential high risks. These contouring decisions were made by the attending physicians based on their clinical judgment, balancing outcomes, and potential toxicities [12,27]. Advanced online adaptation techniques, such as MR-LINAC and Ethos, hold promise for further enhancing treatment procedures and reducing radiation to normal tissues, potentially allowing for reduced target margins.

The beam-on-time for IMPT was highly promising compared to the other techniques. This is due to the current synchrotron proton beam plans being delivered using discrete spot scanning and multi-energy extraction [73]. Discrete spot scanning involves delivering the specified dose for each spot location in a step-by-step manner. Once the spot dose is delivered, the irradiation stops, and the scanning magnets setting is changed to the next location. The purpose of multi-energy extraction is to reduce the possible energy layer switching time and, thus, significantly reduce the proton dose delivery time. This is achieved by delivering several energy layers in one single spill. In contrast, with the single energy layer extraction scheme, the synchrotron would need to decelerate and accelerate between each energy layer, taking approximately 2 s. The energy layer switch time is only 0.5 s with multi-energy extraction techniques. Furthermore, with continuous scanning (i.e., raster scanning), the estimated delivery dose rate could increase by up to 30%.

The beam-on-time represents the continuous radiation time without human interaction. For all four modalities in this study, this was based on estimates from the treatment planning systems. GK and CK deliver all radiation shots or beamlets in a single setup session, meaning the beam-on-time was equivalent to the overall delivery time. Although the beam-on-time has been significantly improved for IMPT on the proton ProBeat-FR system, the additional time for couch and gantry rotation, as well as imaging verification, added up to 2 min for each beam switch, extending the overall delivery time. The beam switch for VMAT typically takes around 1 min. Among the modalities, VMAT plans had the most efficient delivery time, followed by IMPT plans. GK treatments are particularly advantageous for small lesions but may require significantly longer delivery times for larger lesions.

Several studies have compared treatment plans across different delivery modalities in the context of stereotactic radiotherapy [74,75,76,77,78,79]. However, most of these studies typically involve comparisons of only 2–3 delivery systems or comparisons of techniques or treatment modes from the same modality. Almost all of them rely on the traditional gradient index to evaluate plans, which provides limited insight into the steepness of dose fall-off near critical structures. To date, there is only one publication similar to our study that compared GK, CK, VMAT, and proton therapy. That study used different planning systems for VMAT and proton plans and employed different delivery systems for proton therapy [78]. In addition, their comparison primarily focused on intracranial cases, where the target border gradient was less crucial than in skull base scenarios, making the steepness of dose gradients less of a priority. This distinction underscores the unique value of our study in addressing the challenges of skull base reirradiation.

Although we introduced the steepest dose gradient for the most challenging skull base reirradiation scenarios, it is evident that this information can also be applied to other SBRT sites with a similar CT intensity range, such as spinal stereotactic radiotherapy, where strict dose constraints are essential. Moreover, this gradient information provides a deeper understanding of the potential dose distribution of radiotherapy and its impact on critical structures nearby. It can further serve as a valuable tool in decision-making, helping to determine whether radiotherapy or surgery alone or in combination with other treatment modalities is the best approach to achieve optimal cancer control while maintaining a better quality of life post-treatment [4,5,7,80].

While all four of these advanced external beam radiotherapy modalities are suitable for skull base SBRT, the choice depends on several factors beyond the proximity of tumors to critical structures. These factors include patient conditions, availability of techniques, treatment costs, insurance coverage, and more. Proton therapy is often more expensive and less widely available compared to photon-based techniques. GK and CK are dedicated SRS/SBRT modalities, while LINAC machines are versatile, efficient, and widely used for treating various tumors. GK is more suitable for small intracranial lesions, whereas CK and VMAT plans are superior for irregularly shaped tumors. The integration of other modern techniques, such as MRI [51], with these modalities can further enhance treatment outcomes. Additionally, the choice is typically guided by a multidisciplinary team, including radiation oncologists, medical physicists, neurosurgeons, and other specialists.

The limitations of this study include the lack of clinical outcome data to demonstrate the benefit of sharp dose fall-off, the absence of proton aperture to further improve dose gradient and limit dose spread, and a potentially small sample size, which did not capture the effects of tumor shape or multiple lesions on the border gradient metric. Severe toxicities following skull base reirradiation, such as bone or soft tissue necrosis, carotid artery bleeding, cranial nerve damage, and others, could potentially be reduced or even avoided if these critical structures are carefully contoured and spared in the treatment plans using the knowledge of border gradients from this study. At the time of this manuscript, an aperture for head and neck stereotactic radiotherapy on the ProBeat-FR system has not been developed at our institution. However, other institutions have implemented this technique [61,81], demonstrating its advantages. The outcome following skull base reirradiation SBRT and the development of SBRT-specific apertures will be the focus of our future research.

## 5. Conclusions

External beam stereotactic radiotherapy plays an essential role in skull base reirradiation, as it requires steep dose gradients to protect nearby normal structures. The four advanced modalities evaluated in this study demonstrated their suitability for this challenging task. The treatment plans achieved comparable target coverage and dose conformity across the four techniques while meeting similar clinical objectives for protecting adjacent critical structures. The IMPT and VMAT plans demonstrated superior target dose uniformity, whereas the GK plans showed significant inhomogeneity.

Based on the steepest border gradient, GK plans achieved the fastest dose fall-off at the target-OAR border, with a 50% dose drop occurring in approximately 3 mm, compared to around 4 mm for IMPT, CK, and VMAT plans. This border gradient can provide essential guidance during the treatment planning process by defining achievable planning goals to balance effective tumor control with reduced toxicities. This is particularly crucial in situations where nearby OAR tolerance is critical, such as in skull base reirradiation SBRT. The volume gradient showed comparable dose spread-out within 50% prescription isodose lines among the four techniques, whereas IMPT plans demonstrated significantly reduced dose spread into low-dose regions, which is beneficial for minimizing unnecessary radiation exposure to healthy tissues.

## Figures and Tables

**Table 1:** Representative external beam radiation therapy modalities for SBRT settings.

Modalities and Models	Leksell Gamma Knife Perfexion/ICON^TM^	CyberKnife M6/S7	TrueBeam STx	Proton ProBeat-FR
Manufacturers	Elekta	Accuray	Varian	Hitachi
Radiation source/energy	192 sealed Co-60 sources (1.17 MeV and 1.33 MeV)	6 MeV photons on robotic arm	6 MeV, 10 MeV, 6 MeV FFF, 10 MeV FFF Photon on C arm	72.5 MeV–221.8 MeV proton
Mechanical and radiation accuracy	Sub-millimeter	Sub-millimeter	Sub-millimeter	Sub-millimeter
Collimation	Eight-sector crown-shaped collimator	Fixed cone, Iris collimator, InCise MLC	Jaw, high-definition MLC	Aperture, focused collimator
Maximum field size	1.6 cm (shot size)	Fixed cone, Iris collimator: 6 cm MLC: 12 cm × 10 cm	Jaw: 40 cm × 40 cm MLC: 22 cm × 40 cm	30 cm × 40 cm
Beam delivery	Combination of 4, 8, and 16 mm beams (shots)	Beamlets from hundreds of unique angles	Fixed-angle IMRT beams, VMAT	Passive scattering, Spot scanning (IMPT, spot size ~0.5 cm)
Dose rate	2.0 Gy/min (before source change)—3.6 Gy/min (new source)	400–1000 MU/min	400–600 MU/min (6 MeV, 10 MeV photon), 1400 MU/min (6 MeV FFF), 2400 MU/min (10 MeV FFF photon)	480 MU/min **
Onboard imaging *	CBCT (ICON) [29,30]	kV imagers	2D kV/MV and CBCT, 4D CBCT	2D KV and CBCT
Motion management	ICON: high-definition motion management [31,32]	Synchrony respiratory tracking system [33]	External gating system	External gating system
6 DoF setup/motion correction	6 DoF treatment plan adaptation	6 DoF delivery arm	6 DoF couch	6 DoF couch
Commissioning and quality assurance	Petti 2021 (TG 178) [34], Hu 2022 [35]	Sharma 2007 (TG 135) [36], Dieterich 2011 [37]	Klein 2009 (TG 142) [38], Hanley 2021 (TG 198) [39]	Arjomandy 2019 (TG 185) [40], Farr 2021 [41]

DoF: Degree of Freedom; VMAT: volumetric modulated arc therapy; IMRT: intensity-modulated radiation therapy; IMPT: intensity-modulated proton therapy; SBRT: stereotactic body radiation therapy; TG: AAPM Task Group; FF: flattening filter; FFF: flattening filter free; CBCT: cone-beam computed tomography; MLC: multi-leaf collimator. * External imaging systems, such as X-ray imaging (BrainLab’s Exactrac), CT-on-rail, and surface imaging (BrainLab’s Exactrac Dynamic, Vision RT’s Align RT, C-Rad, etc.), can be integrated into radiation delivery systems, as is currently seen with the TrueBeam STx and ProBeat. ** ≥1.25 Gy/min with discrete scanning for the following settings: range: 20 g/cm^2^; target volume: 1 L; and dose: 2 Gy).

**Table 2:** Representative treatment planning systems for RT modalities in Table 1.

Treatment Planning Systems	Leksell GammaPlan	Accuray Precision	RayStation—IMRT/VMAT	RayStation—Proton
Manufacturers	Elekta	Accuray	RaySearch	RaySearch
Planning image	CT (pre-RT), MRI	CT	CT	CT
Isocenter(s) per prescription	Non-isocentric	Non-isocentric	Isocentric	Isocentric
Dose calculation engine	TMR10, convolution	Ray Tracing, FSPB (MLC), Monte Carlo (MLC)	CC Convolution, Monte Carlo	Monte Carlo
inhomogeneity correction	Yes, in convolution when using CT and tumor < 2 cm distance from skin	Yes	Yes	Yes
Optimization	Traditional inverse planning, LDO optimizer [54,55]	VOLO optimizer [56]	DMPO, MCO, robust optimization	DMPO, MCO, robust optimization
Adaptative planning	No	Yes, through PreciseART	Yes	Yes

CC: collapsed cone; LDO: lightning dose optimizer; DMPO: direct machine parameter optimization; MCO: multi-criteria optimization; FSPB: finite-size pencil beam.

**Table 3:** Detailed treatment site, target volume, and prescription for patients treated with SBRT.

Patient	Site	Anatomical/Clinical Region	Target Volume (cm^3^)	Prescription (Gy)	Number of Fractions
1	Petroclival Occiput	Posterior Cranial Fossa	36.4	45	5
2	Petroclival Occiput	Posterior Cranial Fossa	36.4	24	3
3	Petroclival Occiput	Posterior Cranial Fossa	29.8	21	3
4	V3/Ovale	Central Skull Base	29.6	45	5
5	Clivus	Central Skull Base	26.1	45	5
6	Ethmoid/Cribiform	Anterior Cranial Fossa	25.7	45	5
7	Nasopharynx	Retropharynx	21.6	45	5
8	Cavernous Sinus	Central Skull Base	20.7	45	5
9	Retropharyngeal Node	Retropharynx	16.3	45	5
10	Cavernous Sinus	Central Skull Base	15	27	3
11	Cavernous Sinus	Central Skull Base	10.5	21	3
12	Petroclival	Posterior Cranial Fossa	9.2	24	3
13	Retropharyngeal	Retropharynx	9	45	5
14	Retropharyngeal	Retropharynx	7.4	45	5
15	Dura	Intracranial	2.6	24	3
16	Petroclival	Posterior Cranial Fossa	2.1	21	3

**Table 4:** Clinical goals and dose constraints used in SBRT plans for skull base reirradiation.

Structures	Clinical Goals/Dose Constraints	
PTVs	V100% > 95%	
	Dmax < 120%	
OARs	No hot spot if in target, as low as reasonably achievable if outside of or away from target	
	21–27 Gy/3 fractions	40–45 Gy/5 fractions
Brainstem	Dmax < 10 Gy	Dmax < 13 Gy
Spinal cord	Dmax < 9 Gy	Dmax < 12 Gy
Optic apparatus	Dmax < 9 Gy	Dmax < 12 Gy
Carotids	Dmax < 20 Gy	Dmax < 30 Gy
Cochlea	Dmax < 21 Gy	Dmax < 21 Gy
Temporal lobe	Dmax < 18 Gy V12 Gy < 3 cm^3^	Dmax < 27 Gy V18 Gy < 3 cm^3^

SBRT: stereotactic body radiation therapy; PTV: planning target volume; OAR: organ at risk; V100: volume receiving 100% of prescription dose; VxGy: volume receiving x Gy; Dmax: maximum dose.

**Table 5:** Dosimetric comparison of treatment plans for four external beam RT systems based on 16 skull base SBRT patients.

Technique	Primary Target Coverage (%)	PCI	HI	Beam-on-Time (min)	Delivery Time (min)	SBG @90%Rx (%/mm)	SBG @50%Rx (%/mm)
CK	98	0.68	0.24	24.3 *	24.3	12.8	13.9
GK	96.8	0.64	0.56	71.3 *	71.3	20.9	16.6
IMPT	97.9	0.65	0.09	2.2	12.1	12.8	14.1
VMAT	98.1	0.69	0.08	4.4	6.1	10.2	12.5

CK: CyberKnife; GK: Gamma Knife; IMPT: intensity-modulated proton therapy; VMAT: volumetric modulated arc therapy; PCI: Paddick conformity index; HI: homogeneity index; SBG: steepest border gradient. * Based on treatment time reported in the treatment planning system.

## Data Availability

The data presented in this study are available on request from the corresponding author.
